# Differential gene expression in chronic wasting disease‐positive white‐tailed deer (*Odocoileus virginianus*)

**DOI:** 10.1002/ece3.5724

**Published:** 2019-10-30

**Authors:** Emma K. Trone‐Launer, Jun Wang, Guoqing Lu, Nohra E. Mateus‐Pinilla, Paige R. Zick, James T. Lamer, Paul A. Shelton, Christopher N. Jacques

**Affiliations:** ^1^ Department of Biological Sciences Western Illinois University Macomb IL USA; ^2^ Key Laboratory of Freshwater Fisheries Germplasm Resources Ministry of Agriculture Shanghai Ocean University Shanghai China; ^3^ Department of Biology and School of Interdisciplinary Informatics University of Nebraska Omaha Omaha NE USA; ^4^ Illinois Natural History Survey—Prairie Research Institute University of Illinois Urbana‐Champaign Champaign IL USA; ^5^ Illinois River Biological Station Illinois Natural History Survey Havana IL USA; ^6^ Illinois Department of Natural Resources Springfield IL USA; ^7^ Present address: Illinois Department of Natural Resources Coffeen IL USA

**Keywords:** chronic wasting disease, differential gene expression, Illinois, *Odocoileus virginianus*, RNA‐Seq, white‐tailed deer

## Abstract

Chronic wasting disease (CWD) is a transmissible spongiform encephalopathy (TSE) that affects cervid species throughout North America. We evaluated gene expression in white‐tailed deer collected by Illinois Department of Natural Resource wildlife managers during annual population reduction (e.g., sharpshooting) and disease monitoring efforts throughout the CWD‐endemic area of northcentral Illinois. We conducted comparative transcriptomic analysis of liver and retropharyngeal lymph node tissue samples between CWD‐positive (*n* = 5) and CWD‐not detected (*n* = 5) deer. A total of 74,479 transcripts were assembled, and 51,661 (69.36%) transcripts were found to have matched proteins in NCBI‐NR and UniProt. Our analysis of functional categories showed 40,308 transcripts were assigned to at least one Gene Ontology term and 37,853 transcripts were involved in at least one pathway. We identified a total of 59 differentially expressed genes (DEGs) in CWD‐positive deer, of which 36 and 23 were associated with liver and retropharyngeal lymph node tissues, respectively. Functions of DEGs lend support to previous relationships between misfolded PrP and cellular membranes (e.g., STXBP5), and internal cellular components. We identified several genes that suggest a link between CWD and retroviruses and identified the gene ADIPOQ that acts as a tumor necrosis factor (TNF) antagonist. This gene may lead to reduced production of TNF and impact disease progression and clinical symptoms associated with CWD (i.e., wasting syndrome). Use of candidate genes identified in this study suggests the activation of endogenous processes in CWD‐positive deer, which in turn may enable earlier detection of the disease.

## Graphical abstract

We evaluated gene expression in white‐tailed deer collected by the Illinois Department of Natural Resources during annual population reduction and disease monitoring efforts throughout the CWD‐endemic area of northcentral Illinois. We identified 59 differentially expressed genes (DEGs) in CWD–infected deer LV (*n* = 36) and RPLN (*n* = 23) tissues, of which 28 genes were up‐regulated and 31 down‐regulated. Use of candidate genes identified in this study suggests the activation of endogenous processes in CWD‐positive deer, which in turn may enable earlier detection of the disease.

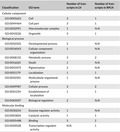

## 1 INTRODUCTION

Transmissible spongiform encephalopathies (TSEs) are fatal neurodegenerative prion diseases (proteinaceous infectious particle; Prusiner, 1982) that infect humans (Creutzfeldt‐Jakob disease, kuru; Brown, 2013), mink (transmissible mink encephalopathy; Hartsough & Burger, 1965), sheep (scrapie; Prusiner, 1989), cattle (bovine spongiform encephalopathy; Hope et al., 1988), and cervids (chronic wasting disease [CWD]; Williams & Young, 1980). Prion diseases are caused by conversion of naturally occurring protease‐sensitive, cellular prion protein (PrP^c^) into a conformationally altered isoform (PrP^Sc^) of prions (Griffith, 1967; Prusiner, 1982). These abnormal prions, or amyloids, accumulate in the central nervous system and peripheral lymphoid tissues (Caughey, Race, & Chesebro, 1988; Kimberlin & Walker, 1982) of a host, which are detergent‐insoluble and partially proteinase K‐resistant (Pan et al., 1993; Prusiner, 1989). Post‐translational alterations from PrP to PrP^Sc^ have been implicated as the causative factor leading to infection (Prusiner, 1989).

Chronic wasting disease (CWD) is of considerable interest and concern to wildlife managers throughout North America (Williams, Miller, Kreeger, Kahn, & Thorne, 2002). Due to the potential risk of transmission, CWD poses a possible threat to domestic species such as cattle (Basu et al., 2012) and swine (Moore et al., 2017), and cannot be ruled out as a potential risk to human health (Waddell et al., 2018). Risk of cross‐species transmission is increased by the ability of prions to affect captive and free‐ranging animals (Williams et al., 2002) as captive wildlife are more likely to come in contact with domestic species. Prevalence rates as high as 50% in free‐ranging herds and 90% in captive herds have been documented (Haley & Hoover, 2015), though prions are difficult to diagnose in live cervids (Cheng et al., 2016) due to the logistical challenges of collecting diagnostic samples. Tonsil, lymphoid, and third eyelid tissues may be biopsied to confirm CWD infection in live animals; however, these tissue biopsies would require sedation or anesthesia in accordance with Institutional Animal Care and Use Committee (IACUC) protocols, and are impractical in free‐ranging herds (Haley & Richt, 2017). Clinical signs may help in visual diagnoses of positive animals, but are only apparent in final stages of disease progression (i.e., months to years after initial infection; Gilch et al., 2011; Williams, 2005), and may be the result of other chronic disease processes.

Little is known about prion transmission in native hosts (Saunders, Bartelt‐Hunt, & Bartz, 2012). Some research suggests underlying mechanisms of PrP^Sc^ formation may be dependent on the type of prion disease (i.e., infectious, sporadic, genetic; Harris, 1999). Chronic wasting disease is transmitted primarily through direct contact between positive and susceptible animals via oral and mucosal membranes (Safar et al., 2008). Contact with prions in the environment via horizontal transmission (Saunders et al., 2012) and mother‐to‐offspring vertical transmission (Nalls et al., 2013; Selariu et al., 2015) contribute to the rate of disease spread. Furthermore, prions are stable enough to withstand environmental changes such as ultraviolet radiation, freeze–thaw cycles, and bacterial and fungal enzymes (Gilch et al., 2011) and persist for at least 1 year (Kuznetsova, Cullingham, McKenzie, & Aiken, 2018; Wyckoff et al., 2016) in soil in the absence of CWD‐positive deer (Johnson et al., 2006).

A single prior study identified gene expression changes in CWD‐positive Rocky Mountain elk (*Cervus elaphus*) using microarray analysis and predetermined transcripts (Basu et al., 2012). This study provided evidence for the involvement of genes assigned to functional groups associated with biological regulation, metabolic process, and cellular process. Moreover, Basu et al. (2012) identified novel genes and numerous pathways that contributed to infection, including calcium signaling, apoptosis and cell death, immune cell trafficking, and inflammatory response. However, confinement to known transcripts is a disadvantage of traditional microarray studies. Studies addressing hypothesis‐driven questions related to the role of specific genes in facilitating or reducing disease infection in white‐tailed deer (*Odocoileus virginianus*; hereafter deer) are difficult to conduct given the limited availability of annotated deer genomes and transcripts currently available in the literature. Furthermore, researchers have evaluated the potential for CWD resistance in relation to sequence polymorphisms (Brandt et al., 2015; Kelly et al., 2008) and potential genetic risk factors (Matsumoto, Samuel, Bollinger, Pybus, & Coltman, 2013), but have not previously examined differentially expressed genes in CWD‐infected and noninfected deer.

Next‐generation sequencing (NGS) allows for discovery of novel transcripts in a more rapid and comprehensive method than other current technologies available (Mardis, 2008) at comparatively low costs (Metzker, 2010). Discovery of novel genes using NGS does not require a priori knowledge of genes that may be present, thus mitigating ascertainment bias. Thus, a need exists for NGS application (Basu et al., 2012) and discovery of novel transcripts to further gene expression studies in all TSE‐impacted species. At the initiation of this research, transcriptome‐level gene expression evaluation in free‐ranging deer using ribonucleic acid (RNA)‐sequencing technology had not previously been conducted.

Regardless of protein polymorphisms, liver tissues from CWD‐positive and clinically affected deer are CWD immunohistochemistry (IHC)‐negative, while retropharyngeal lymph nodes are CWD IHC‐positive (Otero et al., 2019). Therefore, we sought to identify differentially expressed genes in liver, which produces proteins involved in the innate immune response (Gao, Jeong, & Tian, 2008), and retropharyngeal lymph nodes, which are sites of prion accumulation (Williams, 2005), from CWD IHC‐positive and CWD IHC‐not detected (hereafter CWD‐positive and CWD‐ND, respectively), free‐ranging deer. Our study may contribute to an increased understanding of molecular mechanisms involved in the pathology and replication of CWD in cervid species. To our knowledge, this is the first study evaluating gene expression in CWD using NGS to identify novel transcripts.

## 2 MATERIALS AND METHODS

### 2.1 Tissue extraction

From January to March 2015, liver, obex, and retropharyngeal lymph node samples were collected from 380 free‐ranging adult (>1.5 years old) deer (Severinghaus, 1949) euthanized by Illinois Department of Natural Resources wildlife managers during annual population reduction and disease management efforts throughout the CWD‐endemic area of northcentral Illinois (Manjerovic, Green, Mateus‐Pinilla, & Novakofski, 2014; Mateus‐Pinilla, Weng, Ruiz, Shelton, & Novakofski, 2013). Following euthanasia, deer were transported to central processing locations within 6 hr of death, at which time tissues were rinsed using double‐distilled water (ddH_2_O) and any blood removed prior to collection. At the time of necropsy, liver and retropharyngeal lymph node biopsy tissue samples were extracted using 6‐mm Miltex surgical biopsy punches (Ref. Num. 33–36). For each animal, liver tissue samples were collected at the approximate center of the right anterior section of the right lobe. We randomly selected the right or left retropharyngeal lymph node for sampling, and collected tissue from the approximate center of the node. Biopsy punches were placed into 1.5‐ml centrifuge tubes and stored in 1.5 ml RNAlater (Thermo Fisher Scientific, Cat. No. AM7020) per the manufacturer's recommendations (Qiagen, Inc.). Tissue samples were refrigerated for 24 hr at 2°C after which they were placed in a freezer (−10°C) on site. Each week, we transported biopsy samples on dry ice to Western Illinois University, at which time they were stored at −20°C until transported to the Core Genomics Laboratory at University of Illinois Chicago for sequencing.

Wildlife managers from the Illinois Department of Natural Resources submitted CWD diagnostic samples (i.e., retropharyngeal lymph nodes, obex) to the Animal Disease Laboratory in Galesburg, Illinois, USA, for disease testing via immunohistochemistry (IHC). We used test results to select CWD‐positive and CWD‐ND deer from our biopsy sample collection. We conducted RNA integrity analysis to determine suitable samples for sequencing. We paired CWD‐positive suitable samples with randomly selected CWD‐ND individuals of similar locations and age classes (i.e., adults). None of 380 deer sampled showed clinical signs; thus, we assumed that if they were CWD‐positive, they were in similar stages of disease progression (Williams, 2005). The health status of sampled free‐ranging deer was unknown as they had not been tested for other diseases (e.g., epizootic hemorrhagic disease, tuberculosis). From the 380 deer sampled, we selected 10 adult deer for this study. Inclusion criteria were based on integrity of RNA samples, age, sex, and having CWD test results from both obex and retropharyngeal lymph nodes. We used tissue biopsy samples from 10 (5 CWD‐positive [2 males and 3 females; treatment group] and 5 CWD‐ND [3 males and 2 females; control group]) adult free‐ranging deer for RNA‐Seq analyses. All five CWD‐positive deer were IHC‐positive in obex and retropharyngeal lymph nodes.

### 2.2 RNA extraction and sequencing

Using a Qiagen RNeasy Mini Kit (Cat. No. 74104), we extracted RNA from each sample according to the manufacturer's instructions. We examined RNA integrity and quantity using a NanoDrop 1000 and a 2200 TapeStation system using RNA ScreenTape (Agilent, Cat. No. 5067‐5576). A total of 5 μg RNA with an RNA integrity number (RIN) > 7 was used for RNA‐Seq library construction. Additionally, a complementary deoxyribonucleic acid (cDNA) library was prepared with the TruSeq Stranded mRNA LT Sample Prep Kit—Set A (Illumina, Cat. No. RS‐122‐2101) and amplified using polymerase chain reaction (PCR), specifically the Illumina HiSeq 2500 Sequencing System with a HiSeq SBS sequencing kit (Illumina Inc.). Resulting paired‐end reads were 100 base pairs in length and sequenced on one lane from each end for 101 cycles. We generated and demultiplexed FASTQ data files with the bcl2fastq v1.8.4 Illumina Conversion Software (Illumina Inc.). We used RNA‐Seq to analyze liver and retropharyngeal lymph node tissue samples from CWD‐positive and CWD‐ND deer. Low integrity RNA samples (i.e., RIN < 7.0) were excluded from RNA‐Seq analyses.

### 2.3 De novo assembly

Raw sequencing reads were trimmed by Trimmomatic software (Bolger, Lohse, & Usadel, 2014) to remove low‐quality sequencing reads before assembly. Reads with an average quality score below 15 in a 4 base pair sliding window, and reads with quality below 5 at the beginning and end were filtered. After trimming, cleaned reads were used for the reference transcriptome assembly based on Trinity version 2.06 with paired‐end mode (Grabherr et al., 2011). Transcripts from liver and retropharyngeal lymph node tissues were separately assembled de novo. To obtain a comprehensive reference transcriptome, the two assemblies were merged and redundant transcripts were filtered by CD‐HIT software with default parameters (Li & Godzik, 2006). To filter out misassembled transcripts and transcripts with low expression, raw sequenced reads were mapped to assembled reference transcriptomes using Bowtie 1.0.0 (Langmead, Trapnell, Pop, & Salzberg, 2009). Then, transcript abundance, fragments per kilobase per transcript per million mapped reads (FPKM), values were calculated using RNA‐Seq by expectation maximization (RSEM) software (Li & Dewey, 2011), and transcripts with FPKM < 1 were filtered out (Li & Godzik, 2006). Filtered transcripts were used as the deer reference transcriptome for downstream analysis (Grabherr et al., 2011).

### 2.4 Transcriptome annotation

Assembled transcriptomes were annotated using BLASTX against NCBI‐NR and UniProt protein databases, with a cutoff *E*‐value of <1e^−6^. We imported BLASTX results into BLAST2GO software (Conesa et al., 2005), and Gene Ontology (GO) terms, Enzyme Commission numbers, and Kyoto Encyclopedia of Genes and Genomes (KEGG) pathways were annotated by BLAST2GO software. Protein‐coding DNA sequence region was predicted using TransDecoder implemented in Trinity software (Haas et al., 2013). Sequences with a corresponding protein length greater than 100 were retained for further analysis.

### 2.5 Differential gene expression analysis

Clean reads generated from liver and retropharyngeal lymph node tissues from CWD‐positive and CWD‐ND groups were mapped back to our assembled reference transcriptome separately, and fragments per kilobase of exon model per million fragments mapped values were calculated by RSEM software for each individual deer (Li & Dewey, 2011). The resulting data matrix that contained FPKM expression values for liver and retropharyngeal lymph node tissues of each individual was generated by “rsem‐generate‐data‐matrix” script. This data matrix with FPKM values was imported into edgeR 2.14 (Robinson, McCarthy, & Smyth, 2010) to create a pairwise comparison between CWD‐positive and CWD‐ND deer, and identify differentially expressed genes (DEGs) with fold change > 2^2^ (log fold change = log 2 [CWD‐positive FPKM/CWD‐ND FPKM]) and a *p*‐value < .001 for false discovery rate. We presented expressed genes with a false discovery rate ≤ 0.001. Differential expression analyses were conducted between liver samples from CWD‐positive versus CWD‐ND deer, and between retropharyngeal lymph node samples from CWD‐positive versus CWD‐ND deer. We defined an up‐regulated gene as a gene that was differentially expressed in CWD‐positive deer as compared to a CWD‐ND deer, and a down‐regulated gene as a gene that was differentially expressed in CWD‐ND deer as compared to a CWD‐positive deer based on log fold change and false discovery rate results. Genes were analyzed using Gene Ontology (GO) and the Kyoto Encyclopedia of Genes and Genomes. Gene Ontology enrichment analysis of the DEGs detected was conducted by DAVID function annotation tool with Fisher's exact test *p*‐value ≤ .05 (Huang, Sherman, & Lempicki, 2008), to classify DEGs that were molecularly validated based on cellular components, biological processes, and molecular functions.

### 2.6 Gene validation

Real‐time quantitative PCR (qRT‐PCR) was conducted to validate the DEGs identified by RNA‐Seq. Two assays were designed for each region using PrimerQuest Tool from Integrated DNA Technologies, Inc. A total of 40 assays were used, with one assay repeated twice. Additionally, Flex Six BioMark chip (Fluidigm, Inc.) and Eva Green RT‐PCR (Bio‐Rad Laboratories, Inc.) assays were used. Samples were treated with DNase I (Zymo DNase I set, E1010) followed by column purification (i.e., Qiagen RNeasy Micro, Qiagen Cat. ID 74004). Samples were analyzed using a 2200 TapeStation (Thermo Scientific, Cat. No. 4368814) to verify removal of gDNA. Conversion of RNA to cDNA was accomplished using 1 μg of total RNA per reaction and a High Capacity cDNA Reverse Transcription Kit from Applied Biosystems. Complementary DNA was target‐specific preamplified according to a gene expression preamp protocol (Fluidigm, Inc.). We used 12 amplification cycles in the thermal cycling step. Final products were diluted fivefold, and each sample was analyzed in three technical replicates and five biological replicates (i.e., 5 CWD‐positive and 5 CWD‐ND). BioMark reactions were set up as per Fluidigm's quick reference protocol (Fluidigm, Inc.). We performed qRT‐PCR cycling and signal acquisition on the BioMark System and analyzed data using Fluidigm qRT‐PCR analysis software (Spurgeon, Jones, & Ramakrishnan, 2008). We further classified DEGs as passing validation when all above parameters were met and amplification plots showed a clear exponential phase and saturation plateau and no residual primer dimers. We classified DEGs as failing validation when the previously mentioned parameters were not met, which led to no or aberrant amplification plots and melt curves. We classified DEGs as interpret with caution for a variety of reasons (e.g., remaining primer dimers, differing temperature peaks, or failed primers; Table 3) per the protocol of the Core Genomics Lab at the University of Chicago to aid in downstream biological interpretation and prioritization.

## 3 RESULTS

We generated 488,145,350 (243,310,654 from liver tissue and 244,834,696 from retropharyngeal lymph node tissue) clean pair‐end reads through RNA‐sequencing. Mean assembled transcripts ranged in size from 400 base pairs to >5,000 base pairs. After removal of transcripts with low expression and redundancy, we retained 74,479 transcripts as a reference transcriptome (number of N50 transcripts = 14,877, N50 length = 3,204 base pairs, mean length = 2,108 base pairs). In addition, 51,647 transcripts were assigned genes in NCBI‐NR and 47,292 transcripts were assigned genes in UniProt; 51,661 (69.36%) were assigned to a known gene. *Ovis aries*, *Bos taurus*, *Bubalus bubalis*, *Bos mutus*, and *Capra hircus* (Table 1) were the top species associated with BLAST hits against the NCBI‐NR database. These species accounted for 49.08% (20,980) of the BLAST hits, whereas the “others” category accounted for 8,696 BLAST hits. The “others” category accounted for all other organisms (>290 species) annotated beyond the top 29 species (Table 1). There were 7,899 genes shared between liver and retropharyngeal lymph node tissues.

**Table 1:** Species distribution of top BLAST results of de novo‐assembled white‐tailed deer transcripts against NCBI‐NR database

Species	BLAST top‐hits
*Ovis aries*	8,904
Others^a^	8,696
*Bos taurus*	7,361
*Bubalus bubalis*	5,837
*Bos mutus*	4,831
*Capra hircus*	2,951
*Pantholops hodgsonii*	2,771
*Bison bison*	2,535
*Homo sapiens*	1,150
*Sus scrofa*	582
*Mus musculus*	513
*Balaenoptera acutorostrata*	508
*Camelus ferus*	442
*Physeter catodon*	398
*Orcinus orca*	349
*Myotis brandtii*	291
*Equus przewalskii*	278
*Equus caballus*	277
*Pteropus alecto*	276
*Lipotes vexillifer*	275
*Tursiops truncatus*	272
*Cricetulus griseus*	271
Synthetic construct	252
*Tupaia chinensis*	251
*Rattus norvegicus*	250
*Ursus maritimus*	239
*Ailuropoda melanoleuca*	237
*Chlorocebus sabaeus*	222
*Canis lupus*	221
*Macaca mulatta*	207

a The “others” category accounts for all other organisms (>290) annotated beyond the top 29 species summarized in the table.

We assigned 40,308 transcripts at least one GO term, and 37,853 transcripts were assigned to at least one pathway; we limited reporting of GO terms to those with ≥1,000 assigned transcripts. Gene Ontology analysis identified transcripts successfully mapped to 16 GO biological processes (GO level 2; Table 2). Most (59.0%) transcripts were related to cellular process, metabolic process, single‐organism process, or biological regulation. Additionally, transcripts were mapped to six cellular components and seven molecular functions (GO level 2). Most (60.8%) transcripts assigned to a cellular process were related to cells and organelles. Similarly, most (80.9%) transcripts mapped to molecular functions were related to binding and catalytic activity. Top Kyoto Encyclopedia of Genes and Genomes pathways include purine metabolism, biosynthesis of antibiotics, pyrimidine metabolism, glycerophospholipid metabolism, and phosphatidylinositol signaling system (Table 3).

**Table 2:** Number of Gene Ontology (GO) analysis identified white‐tailed deer transcripts with a cutoff *E*‐value of <1e^−6^ and corresponding protein length greater than 100, successfully mapped to cellular component, biological process, and molecular function classifications

GO number	Classification	No. of transcripts^a^
Cellular component		
GO:0005623	Cell	27,177
GO:0043226	Organelle	21,631
GO:0016020	Membrane	11,980
GO:0032991	Macromolecular complex	9,618
GO:0031974	Membrane‐enclosed lumen	5,757
GO:0005576	Extracellular region	2,021
Biological process		
GO:0009987	Cellular process	27,280
GO:0008152	Metabolic process	24,872
GO:0044699	Single‐organism process	22,464
GO:0065007	Biological regulation	16,474
GO:0050896	Response to stimulus	11,215
GO:0071840	Cellular component organization or biogenesis	8,501
GO:0051179	Localization	8,496
GO:0023052	Signaling	7,363
GO:0032501	Multicellular organismal process	7,242
GO:0032502	Developmental process	6,538
GO:0002376	Immune system process	3,473
GO:0051704	Multi‐organism process	2,741
GO:0040011	Locomotion	1,799
GO:0022610	Biological adhesion	1,762
GO:0022414	Reproductive process	1,165
GO:0040007	Growth	1,157
Molecular function		
GO:0005488	Binding	24,330
GO:0003824	Catalytic activity	16,073
GO:0098772	Molecular function regulator	1,982
GO:0005215	Transporter activity	1,914
GO:0060089	Molecular transducer activity	1,804
GO:0001071	Nucleic acid binding transcription factor activity	1,391
GO:0005198	Structural molecule activity	1,138

a Reporting of GO terms was limited to those with ≥1,000 assigned transcripts.

**Table 3:** Top 40 Kyoto Encyclopedia of Genes and Genomes (KEGG) pathways in the white‐tailed deer de novo assembly transcriptome

Pathway	Sequences in pathway	Number of enzymes
Purine metabolism	843	55
Biosynthesis of antibiotics	841	126
Pyrimidine metabolism	343	33
Glycerophospholipid metabolism	255	31
Phosphatidylinositol signaling system	250	22
Lysine degradation	244	19
Aminoacyl‐tRNA biosynthesis	230	24
Glycolysis/gluconeogenesis	228	26
Glutathione metabolism	199	19
Drug metabolism—cytochrome P450	194	7
Fatty acid degradation	186	15
Tryptophan metabolism	183	21
Glycerolipid metabolism	180	16
Metabolism of xenobiotics by cytochrome P450	178	8
Oxidative phosphorylation	178	7
Pyruvate metabolism	174	20
Amino sugar and nucleotide sugar metabolism	174	35
Glycine, serine, and threonine metabolism	170	31
Carbon fixation pathways in prokaryotes	163	20
Inositol phosphate metabolism	162	21
T‐cell receptor signaling pathway	162	2
Thiamine metabolism	161	4
Valine, leucine, and isoleucine degradation	159	24
Nicotinate and nicotinamide metabolism	157	16
Citrate cycle (TCA cycle)	156	17
Arachidonic acid metabolism	142	17
Sphingolipid metabolism	142	20
Cysteine and methionine metabolism	139	25
Pentose phosphate pathway	125	17
Drug metabolism—other enzymes	122	17
Methane metabolism	120	15
Steroid hormone biosynthesis	117	16
Butanoate metabolism	111	15
Arginine and proline metabolism	108	23
Retinol metabolism	105	10
Alanine, aspartate, and glutamate metabolism	103	24
Starch and sucrose metabolism	102	18
Propanoate metabolism	101	16
Biosynthesis of unsaturated fatty acids	100	8

We identified 59 genes as differentially expressed in CWD‐positive (as compared to CWD‐ND) deer liver and retropharyngeal tissues (Table 4). Among these, 36 were found in liver tissue (16 up‐regulated, 20 down‐regulated) and 23 (12 up‐regulated, 11 down‐regulated) in retropharyngeal lymph node tissue; 29 genes have a known function when compared to UniProt and NCBI databases. Of 59 genes, 33 passed validation, 14 failed, and 12 should be interpreted with caution (Table 4). Function of genes that passed validation includes sodium channel proteins, endogenous retrovirus proteins, and cell death activators. Differentially expressed genes associated with liver and retropharyngeal lymph node tissues included top functions assigned by Gene Ontology associated with cellular membranes, binding, apoptosis, metabolic processes, cellular processes, and catalytic activity (Table 5). Furthermore, we identified several DEGs (i.e., ERVK13‐1, ERVK‐24) which were up‐regulated in the disease state and assigned a Gene Ontology cellular component of plasma membrane.

**Table 4:** Differentially expressed genes in chronic wasting disease‐positive liver (LV) and retropharyngeal lymph node (RPLN) tissues from white‐tailed deer collected in the chronic wasting disease‐endemic area of northern Illinois during annual population reduction, winter 2015

Differentially expressed gene identification^a^	logFC^b^	FDR	CWD‐ positive FPKM^c^	CWD‐ ND FPKM^d^	Annotation
Up‐regulated in CWD‐positive LV					
L_TR43469|c2_g3_i5^e^	3.39	6.12E−06	6.05	0.57	Endogenous retrovirus group k member 25 env poly; ERVK13‐1
L_TR63450|c1_g1_i1^f^	5.28	1.50E−05	6.29	0.16	Uncharacterized protein loc105607204 isoform x1
L_TR45335|c0_g1_i1^g^	2.97	2.34E−05	3.9	0.49	Unknown
L_TR29095|c7_g3_i1^g^	3.24	2.67E−05	4.24	0.44	Sodium channel protein type 11 subunit partial
L_TR56520|c6_g2_i1^g^	4.64	0.001065	2.15	0.08	Unknown
L_TR56520|c6_g3_i2^g^	5.79	0.002325	1.15	0.02	Syntaxin‐binding protein 5; STXBP5
L_TR41343|c0_g1_i2^g^	4.28	0.004595	3.61	0.18	Uncharacterized protein loc102402433 isoform x1
L_TR49285|c0_g1_i1^e^	5.16	0.00801	2.73	0.07	Unknown
L_TR77350|c0_g1_i1^e^	3.98	0.00963	4.46	0.27	Gag protein, ERVK‐24
L_TR47646|c1_g1_i1^f^	2.32	0.028849	4.67	0.92	Unknown
L_TR56520|c6_g1_i1^g^	4.24	0.030398	1.14	0.06	Syntaxin‐binding protein 5
L_TR53215|c2_g3_i1^e^	7.08	0.038974	1.71	0	Dual metabolic roles of gluconeogenesis and glyoxylate detoxification; AGXT
L_TR41343|c0_g1_i3^f^	3.77	0.040655	2.86	0.2	Unknown
L_TR79592|c7_g2_i1^g^	3.36	0.04327	1.8	0.17	Interleukin‐17 receptor a isoform x2
L_TR27390|c2_g1_i1^g^	11.61	0.046946	3.22	0	Tumor necrosis factor antagonist; ADIPOQ
L_TR41343|c0_g1_i1^f^	5.01	0.046946	4.06	0.12	Unknown
Down‐regulated in CWD‐positive LV					
L_TR47259|c0_g1_i1^f^	−9.76	1.50E−05	0.03	34.22	Uncharacterized protein loc105607204 isoform x1
L_TR10266|c1_g2_i3^e^	−3.83	1.50E−05	1.52	21.59	Acyl‐binding protein; DBI
L_TR8752|c0_g3_i2^g^	−2.05	0.00088	28.5	117.84	Zinc ion binding; Rsp29
L_TR28955|c0_g1_i1^g^	−8.13	0.001462	0.02	10.09	Unknown
L_TR30917|c0_g1_i1	−3.87	0.002742	0.07	1.09	Upf0545 protein c22orf39 homolog isoform x1
L_TR28955|c0_g2_i1^g^	−6.93	0.00801	0.05	8.13	Ubiquinone biosynthesis protein coq4 mitochondrial isoform x5
L_TR29354|c0_g1_i1^g^	−3.05	0.00801	0.51	4.21	Positive regulation of tumor necrosis factor; CCL3
L_TR5337|c0_g1_i1^e^	−4.73	0.00801	0.12	3.34	Unknown
L_TR60746|c4_g1_i1^g^	−4.48	0.00801	0.05	1.3	Uncharacterized protein partial
L_TR74636|c14_g8_i1^e^	−2.02	0.00801	11.5	46.47	Craniofacial development protein 2; TMCO5B
L_TR26826|c4_g1_i1^g^	−2.95	0.009568	0.38	3.06	Endonuclease–reverse transcriptase
L_TR22918|c2_g1_i1^e^	−3.99	0.015372	1.48	23.38	Myomegalin isoform x14
L_TR43779|c0_g1_i2^f^	−7.74	0.015372	0	3.7	Unknown
L_TR62456|c0_g1_i1^g^	−2.65	0.015945	1.74	13.38	Unknown
L_TR69615|c0_g1_i1^g^	−3.65	0.019822	2.1	25.99	Unknown
L_TR3476|c0_g1_i1^g^	−2.61	0.023558	1.05	6.36	Unknown
L_TR46171|c0_g2_i2^g^	−2.84	0.023558	0.51	3.59	Unknown
L_TR15492|c1_g1_i1^f^	−4.05	0.027461	0.13	2.14	Endonuclease–reverse transcriptase, POL; P11369
L_TR26463|c1_g1_i1^g^	−4	0.028849	0.93	15.15	Unknown
L_TR23471|c1_g1_i3^g^	−2.45	0.028849	0.42	2.31	Cell death activator cide‐3 isoform x2; CIDEC
Up‐regulated in CWD‐positive RPLN					
N_TR92656|c3_g7_i1^f^	3.15	9.60E−05	3.82	0.4	Unknown
N_TR181264|c0_g1_i1^g^	5.42	0.000109	4.8	0.1	Low‐quality protein: saoe class i histocompatibility a alpha chain‐like
N_TR87145|c0_g1_i4^g^	7.75	0.000242	2.38	0	Unknown
N_TR101034|c0_g4_i11^f^	2.03	0.002067	4.01	0.99	Unknown
N_TR113697|c0_g2_i1^g^	2.5	0.00294	3.31	0.59	Low‐quality protein: endogenous retrovirus group k member 25, POL; ERVK‐25
N_TR160975|c0_g1_i1^g^	2.02	0.003354	5.01	1.24	Collagen VI acts as a cell‐binding protein; COL6A5
N_TR122168|c0_g4_i1^e^	2.25	0.012216	5.26	1.08	Acts as coactivator in regulation of translation initiation of poly(A)‐mRNA; PAIP1
N_TR108513|c1_g2_i1^g^	2.69	0.016226	3.93	0.58	Unknown
N_TR100013|c5_g1_i1^g^	2.31	0.018102	2.91	0.58	Unknown
N_TR113697|c0_g5_i1^g^	2.45	0.022443	5	0.91	Uncharacterized protein loc104969954 isoform x1
N_TR158607|c0_g2_i2^g^	3.42	0.02443	2.2	0.19	Unknown
N_TR164441|c0_g1_i1^g^	2.46	0.035534	6.64	1.19	Unknown
Down‐regulated in CWD‐positive RPLN					
N_TR151520|c0_g2_i1^f^	−2.13	4.98E−06	6.29	27.09	Nascent polypeptide‐associated complex subunit alpha; NACA
N_TR149140|c6_g1_i2^f^	−8.29	1.23E−05	0	3.72	Unknown
N_TR81328|c8_g2_i1^g^	−8.58	0.000142	0	4.69	Unknown
N_TR81328|c8_g5_i1^f^	−7.3	0.000255	0	2.33	Unknown
N_TR81328|c8_g1_i1^e^	−6.65	0.00079	0	1.69	Unknown
N_TR84619|c1_g1_i3^e^	−2.03	0.002067	1.38	5.52	Uridine‐cytidine kinase 1, UCK1
N_TR93637|c1_g1_i1^g^	−2.32	0.002599	0.59	2.99	Unknown
N_TR41415|c0_g1_i1^f^	−2.51	0.00294	0.26	1.48	Unknown
N_TR43226|c0_g1_i1^f^	−3.67	0.005155	0.4	5.16	Unknown
N_TR164448|c1_g2_i2^e^	−4.39	0.007536	0.14	2.94	Unknown
N_TR97881|c0_g1_i1^g^	−5.73	0.026083	0.16	8.02	May be involved in signal transduction as component of a multimeric receptor complex; Ms4a

Note

We defined an up‐regulated gene as a gene that was differentially expressed in CWD‐positive white‐tailed deer as compared to a CWD‐ND (i.e., not detected) white‐tailed deer, and a down‐regulated gene as a gene that was differentially expressed in CWD‐ND white‐tailed deer as compared to a CWD‐positive white‐tailed deer based on log fold change (FC) and false discovery rate (FDR) results.

a DEGs are those with fold change (log FC) > 2^2^ and a *p*‐value < .001 for false discovery rate (FDR).

b FC = log 2 (CWD‐positive FPKM/CWD‐ND FPKM).

c CWD‐positive FPKM = fragments per kilobase of exon model per million fragments mapped expression values for liver and retropharyngeal lymph node tissues for CWD‐positive white‐tailed deer.

d CWD‐ND FPKM = fragments per kilobase of exon model per million fragments mapped expression values for liver and retropharyngeal lymph node tissues for white‐tailed deer in which CWD was not detected.

e Genes that should be interpreted with caution.

f Genes that failed validation.

g Genes that passed validation.

**Table 5:** Gene ontology (GO) classifications for differentially expressed genes (DEGs) in liver (LV) and retropharyngeal lymph node (RPLN) tissue of chronic wasting disease‐positive white‐tailed deer collected in the chronic wasting disease‐endemic area of northern Illinois during annual population reduction, winter 2015

Classification	GO term	Number of transcripts in LV	Number of transcripts in RPLN
Cellular component			
GO:0005623	Cell	3	1
GO:0044464	Cell part	3	1
GO:0032991	Macromolecular complex	1	N/A
GO:0043226	Organelle	3	1
Biological process			
GO:0032502	Developmental process	2	N/A
GO:0016043	Cellular component organization	1	N/A
GO:0008152	Metabolic process	3	2
GO:0016265	Death	1	N/A
GO:0043473	Pigmentation	2	N/A
GO:0051179	Localization	1	1
GO:0032501	Multicellular organismal process	1	N/A
GO:0009987	Cellular process	3	2
GO:0051234	Establishment of localization	1	1
GO:0065007	Biological regulation	2	N/A
Molecular binding			
GO:0030234	Enzyme regulator activity	1	N/A
GO:0003824	Catalytic activity	1	1
GO:0005488	Binding	3	2
GO:0030528	Transcription regulator activity	N/A	1

## 4 DISCUSSION

### 4.1 PrP misfolding on plasma membranes potentially linked to CWD

Differentially expressed genes assigned to Gene Ontology cellular component plasma membrane may suggest a change occurring in the plasma membrane of CWD animals, in agreement with previous research by Ersdal, Goodsir, Simmons, McGovern, and Jeffrey (2009). Naturally occurring PrP^C^ is attached to the outer surface of the plasma membrane (Peters et al., 2003) and has been shown to be expressed during infection (Linden et al., 2008). Naturally occurring PrP has multiple binding partners involved in cytoskeletal processes (e.g., maintenance, cell growth; Zafar et al., 2011), and its function has been linked to copper homeostasis, oxidative stress, cell survival differentiation, cell signaling, and cell proliferation. Additionally, it has been associated with synaptic function, maintenance, or structure and a regulatory role at central and peripheral synapses (Westergard, Christensen, & Harris, 2007). Functions of PrP and DEG syntaxin‐binding protein 5 (STXBP5) are overlapped. Like PrP, STXBP5 may regulate the ability of presynaptic vesicles to fuse and dock with presynaptic membranes (Bennett, Calakos, & Scheller, 1992) by inhibiting formation of SNARE complexes (i.e., SNAp REceptor; complexes that involve syntaxin, SNAP‐25, and synaptobrevin), which play a role in the release of neurotransmitters (Asuni, Cunningham, Vigneswaran, Perry, & O'Connor, 2008). Furthermore, an interaction critical to the successful conversion of PrP to PrP^Sc^ may take place on the plasma membrane (Caughey, Raymond, Ernst, & Race, 1991). Blocking the specific site of conversion may potentially prevent the misfolding of PrP into PrP^Sc^.

### 4.2 Potential link between CWD and retroviruses

All vertebrate genomes studied include integrated RNA viral sequences known as endogenous retroviruses (ERVs), which were previously considered “junk DNA” (Lee, Jeong, Choi, & Kim, 2013). Several ERV genes, including ERVK13‐1 and ERVK‐24, ERVK‐25, and P11369 (Table 3), were identified in this study, suggesting a potential link between CWD and retroviruses. In addition, at least two DEGs are associated with ERV Gag, the protein responsible for synthesis of structural proteins necessary for the viral core. Proteins Pol and Env, which encode for reverse transcriptase, and proteins of the viral envelope, respectively, also were differentially expressed in CWD‐positive deer (Coffin, Hughes, & Varmus, 1997). Combined with Pro, a virion protease, Gag, Env, and Pol create the backbone of replicating retroviruses (Petropoulos, 1997). Although Pro is not specifically listed in our DEGs, one of the unknown genes may be a form of the protein. Endogenous retroviruses may be activated in prion diseases (Lee et al., 2013), and previous studies suggest retroviruses can serve as cofactors involved in prion diseases (Leblanc et al., 2006) potentially altering endocytic pathways (Ashok & Hegde, 2006). Leblanc et al. (2006) suggested retroviruses may increase prion infectivity by acting as transport vectors in the spread of infective prions throughout an individual. Retroviral Gag was suggested to enhance the release of prion proteins in cellular culture when expressed (Leblanc et al., 2006) as was further demonstrated by Bian et al. (2010) using CWD prions. Additionally, PrP has been suggested to influence retroviral activity as it may act as an antiretroviral, specifically in the spleen after immune stimulation (Lötscher et al., 2007). Prions and retroviral cells may be localized in the same cellular compartments, thus acting as cofactors in infection (Leblanc et al., 2006).

### 4.3 Association between CWD and immune‐related genes

Several differentially expressed genes identified in our study (i.e., ADIPOQ, CCL3; Table 3) are related to tumor necrosis factor (TNF), a cytokine that produces an immune response to help prevent the spread of infection. It induces fever and apoptotic cell death, and inhibits viral replication. Chronic exposure to TNF can lead to shock‐like symptoms including a wasting syndrome (Chu, 2013). It also is important to maintaining follicular dendritic cell networks (Sallusto & Lanzavecchia, 1994). Kitamoto, Muramoto, Mohri, Doh‐Ura, and Tateishi (1991) suggested follicular dendritic cells were important to the replication of prions in lymphoid tissues as early Prp^Sc^ accumulates on these cells. Specifically, ADIPOQ, a TNF antagonist (Masaki et al., 2004), is up‐regulated in liver tissues of positive deer. A monokine, CCL3, is down‐regulated in liver tissues of positive deer and is responsible for positive regulation of TNF production (Ramos et al., 2005). Such regulation of TNF suggests CWD‐positive deer may have down‐regulated TNF production at the time of sampling. However, IHC results were positive in retropharyngeal lymph node and obex, suggesting that CWD‐positive deer were not in the early stages of infection. The presence of TNF genes associated with liver tissue and their absence within retropharyngeal lymph node tissue are to be expected, as the liver is a part of the innate immune system (Gao et al., 2008).

In this study, IHC determined whether a deer was CWD‐positive or CWD‐ND; a CWD‐positive deer was assumed to be far enough (i.e., not recently infected) in disease progression to exhibit an accumulation of prions in the RPLN tissue and obex. Although speculative, it is possible that prior to the IHC detectable stage of CWD infection, an initial increase in TNF production occurs in response to initial infection, which overtime becomes detrimental to deer whose response is to down‐regulate TNF as identified in this study. There was no recorded evidence of declining physical condition in CWD‐positive deer, which may be associated with long incubation periods (2–4 years) and absence of clinical symptoms during early stages of prion infection (Williams, 2005). Nevertheless, during later stages of infection chronic exposure to low concentrations of TNF (Wajant, Pfizenmaier, & Scheurich, 2003) may contribute to the wasting syndrome, depression, and cachexia associated with CWD (Chu, 2013). Additionally, an up‐regulated DEG in liver tissue of positive deer associated with interleukin‐17 (IL‐17; Table 3), is responsible for communication between cells, specifically as an inflammatory response in positive individuals (Huang, Zhang, & He, 2015). Furthermore, other studies have shown interleukin genes to be implicated in prion disease pathogenesis and the innate immune system (Bradford & Mabbott, 2012). Moreover, Meling, Skovgaard, Bårdsen, Heegaard, and Ulvund (2018) reported transcriptional innate immune responses in liver tissues of TSE‐positive animals. Although our study only used 5 CWD‐positive and 5 CWD‐negative deer, further investigation of these genes across known stages of disease progression in a larger sample of infected individuals may lead to a better understanding of the immune response to CWD (or other TSEs) or the identification of additional genes.

### 4.4 Use of candidate genes for early detection

Logistics of collecting and preserving high‐quality tissue samples and transporting samples to laboratory settings for storage make field‐based RNA studies difficult. However, these types of studies are important in eliminating confounding factors (i.e., exposure to artificially high concentrations of prions, inheritance of partial CWD resistance‐conferring PrP polymorphism at greater frequency than in natural settings) induced by captive breeding and evaluating transmission in a natural setting. Animals in captive facilities are exposed to higher concentrations of CWD prions over less space than their free‐ranging counterparts. An increase in the number of CWD‐positive animals in a smaller area may lead to higher infection rates in captive individuals and exposure to higher infectious doses of prions than their free‐ranging conspecifics (Miller & Wild, 2004). A difference in prion concentration may affect gene expression and time of detection, thereby highlighting the importance of examining CWD gene expression in free‐ranging naturally infected individuals. Consequently, a CWD‐positive animal may not exhibit prion concentrations high enough for detection using traditional methods if tested too early in disease progression (Haley, Mathiason, Zabel, Telling, & Hoover, 2009). However, use of gene chips and in situ hybridization may enable researchers to select specific candidate genes as indicators of disease status (Lein, Zhao, & Gage, 2004). Gene expression analyses allow for the detection of genetic responses to stimuli before they are phenotypically visible (Klaper & Thomas, 2004), and use of DEGs identified in this study as candidate genes suggests the activation of endogenous process in CWD‐infected deer that may advance the pathological process.

### 4.5 Validation of unknown DEGs and potential functions

Genes that passed validation and were unannotated are candidates for further study. These genes could have implications for the transmission or replication of infectious prion proteins. Even genes that did not pass validation or that should be interpreted with caution may benefit from testing with additional primer sets. Any potential role of the DEGs discussed in this study should be examined in normal prion proteins. Normal PrP function is ambiguous, and DEGs identified in this study may further the understanding of PrP. While many normal prion protein functions have been described, underlying pathogenesis of TSEs is not well understood as amyloid deposits can be found in outwardly healthy individuals (Diack et al., 2016). It also remains unclear whether conversion of PrP^C^ to PrP^Sc^ leads to a gain of function in PrP^Sc^‐positive individuals (Collins, Lawson, & Masters, 2004) or a loss of function (Samaia & Brentani, 1998). Additionally, DEGs associated with retroviruses warrant further investigation as they may be involved in CWD endocytic cell pathways related to CWD. Future studies should build upon CWD‐associated DEGs identified in this study by examining DEGs in other tissues (i.e., brain stem, blood, rectoanal mucosa‐associated lymph tissue) used in routine disease surveillance (Williams, 2005). Further genetic analyses at a transcriptome level could lead to a greater understanding of naturally occurring prion protein functions and thus aid in the understanding of disease‐causing prion infection and formation mechanisms.

## 5 CONCLUSIONS

Chronic wasting disease has been widely studied; however, many of the underlying mechanisms that influence transmission and disease spread in infected deer are not well understood. Our research highlights several areas for further investigation. Similar to Ersdal et al. (2009), our research suggests a change occurs in the plasma membrane of CWD‐positive deer. Although not explicitly evaluated in this study, this could be due to coinfections with retrovirus or activation of endogenous retrovirus. Furthermore, as Gag, Env, and Pol proteins are differentially expressed, this suggests a link between endogenous retroviruses and CWD as previously presented by Leblanc et al. (2006). Additionally, further investigation of DEGs collected from a wider range of CWD tissues (i.e., obex, blood, tonsils, spleen) may provide greater insight into the mechanisms of disease progression. Investigation of DEGs we have presented may allow for the monitoring of specific genes and their expression, suggesting the activation of endogenous process in CWD‐infected deer.

## CONFLICT OF INTEREST

None declared.

## AUTHOR CONTRIBUTIONS

CNJ and PAS conceived and designed the research; EKT‐L and PAS collected data; EKT‐L, GL, and JW contributed reagents/materials and conducted analyses; EKT‐L, GL, JTL, JW, PRZ, NEM‐P, and CNJ wrote and substantially edited the manuscript.

## Data Availability

RNA‐Seq data are available at the NCBI SRA SRP158695.
